# Supplementary material for: Genetic drift and purifying selection shape within-host influenza A virus populations during natural swine infections
Source: bioRxiv. 2023 Oct 25:2023.10.23.563581. Preprint. [Version 1] doi: 10.1101/2023.10.23.563581 (PMC10634741; doi:10.1101/2023.10.23.563581)
Supplement: 1 [file NIHPP2023.10.23.563581V1-supplement-1.pdf]

bioRxiv preprint doi: https://doi.org/10.1101/2023.10.23.563581; this version posted October 25, 2023. The copyright holder for this preprint (which was not certified by peer review) is the author/funder, who has granted bioRxiv a license to display the preprint in perpetuity. It is made available under aCC-BY 4.0 International license.

Supplementary information captions

Supplementary Tables

Table S1. Nasal wipes collected over the week-long county fair. Columns include vial numbers, pig identification numbers, sampling day, Ct value from the rRT-PCR assay targeting the M segment, infection status based on this assay, and subtype-specific assay results, when performed. A sample was classified as PCR positive for IAV if the Ct value of the qRT-PCR assay targeting the M segment was ≤45.0. Sequence identification numbers of samples that were sequenced are also provided. These unique sequence IDs correspond with those deposited to the NCBI SRA ([pending CDC review, insert bioproject number]).

Table S2. Table of sequenced samples. The first 8 columns of the table include the vial number, pig ID, sampling day, Ct value from the rRT-PCR assay targeting the M segment, and subtype-specific assay results (when available). The next 10 columns of the table include the unique sequence ID from Run 1, the classification of each gene segment into a lineage based on the deep sequencing data, and the classification of the sample into a lineage based on all viral gene segments. The last 10 columns include analogous information and results based on Run 2, when applicable.

Table S3. List of differentiating SNPs (dSNPs) for the six internal gene segments. Each tab lists the gene segment, the dSNP nucleotide site, and the nucleotides present at the corresponding dSNP sites for lineage I and lineage II viruses.

bioRxiv preprint doi: https://doi.org/10.1101/2023.10.23.563581; this version posted October 25, 2023. The copyright holder for this preprint (which was not certified by peer review) is the author/funder, who has granted bioRxiv a license to display the preprint in perpetuity. It is made available under aCC-BY 4.0 International license.

Supplementary Figures

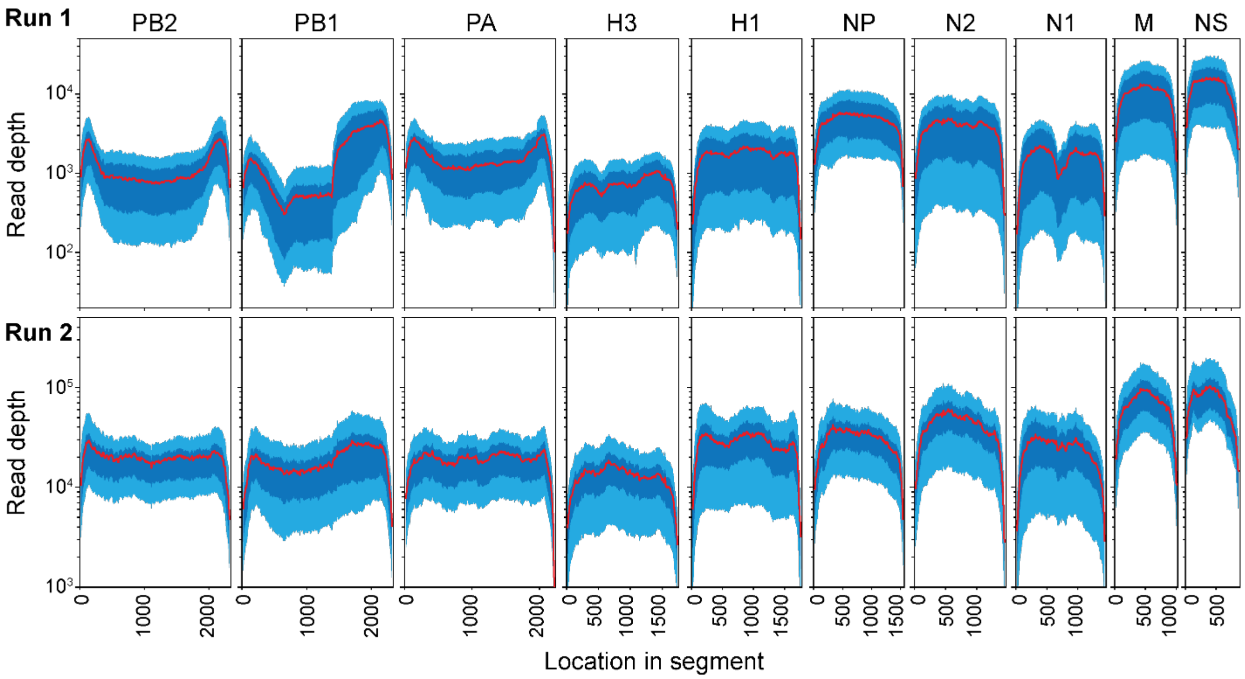

Figure S1. Coverage for sequenced samples. The x-axis shows the location along the IAV genome. The y-axis shows read depth. Coverage for Run 1 and Run 2 samples are shown separately. A sliding window of 200 nucleotide sites was used to calculate mean read depth for each sample at each site. The plotted ranges show median (red), 25th (dark blue), and 75th (light blue) percentiles of read depth across all samples sequenced in Runs 1 and 2.

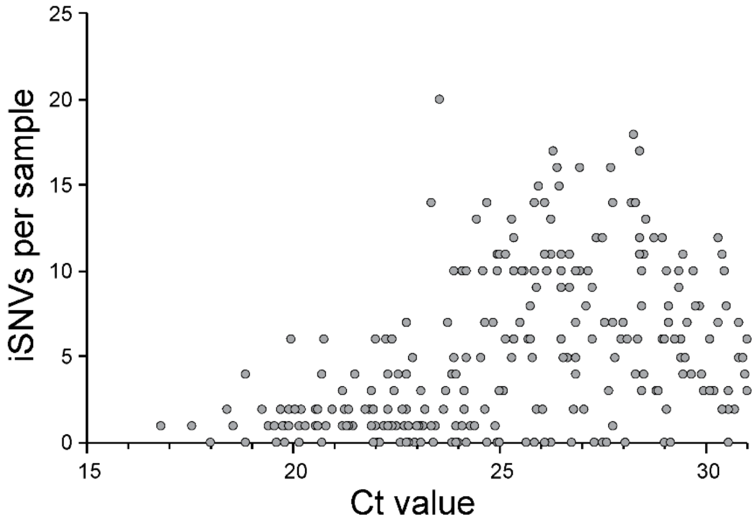

Figure S2. Scatterplot showing the relationship between the rRT-PCR Ct value of a sample and the number of iSNVs detected in the sample. A positive relationship is evident, with higher Ct samples more likely to have a larger number of iSNVs detected.

bioRxiv preprint doi: https://doi.org/10.1101/2023.10.23.563581; this version posted October 25, 2023. The copyright holder for this preprint (which was not certified by peer review) is the author/funder, who has granted bioRxiv a license to display the preprint in perpetuity. It is made available under aCC-BY 4.0 International license.

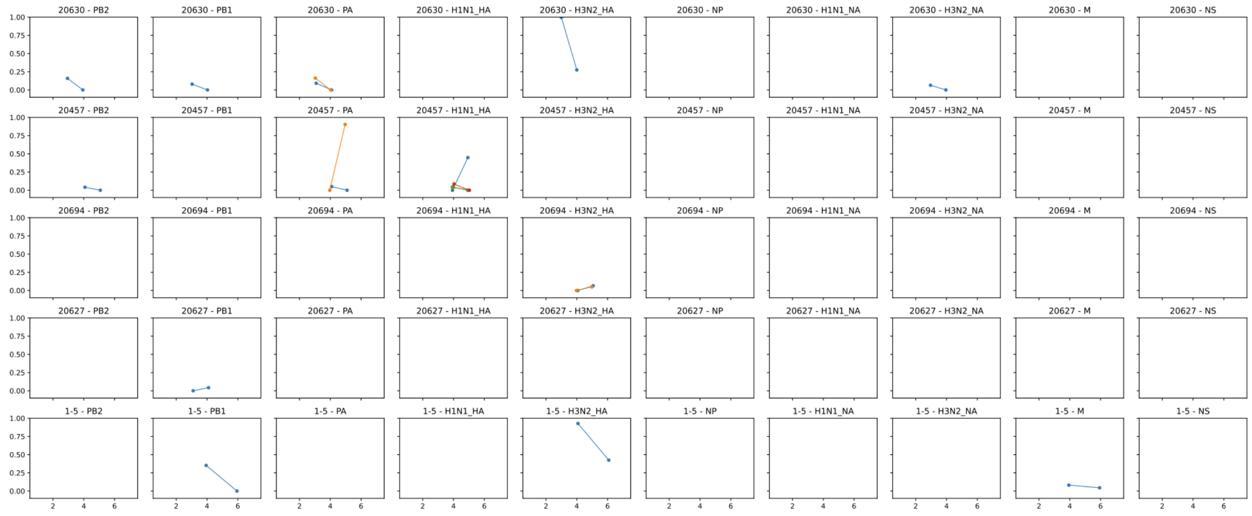

Figure S3. iSNV dynamics within singly infected pigs with two or more sequenced samples. Each row shows iSNV dynamics for a single pig. Columns correspond to the six internal gene segments of IAV, and both lineage I and II HA and NA gene segments. Blank graphs indicate no iSNVs were detected in the reads mapping to each reference sequence. The x-axis specifies the sample day and the y-axis specifies iSNV frequency. ***(Figure above is representative only, see attached PDF for full length figure).***

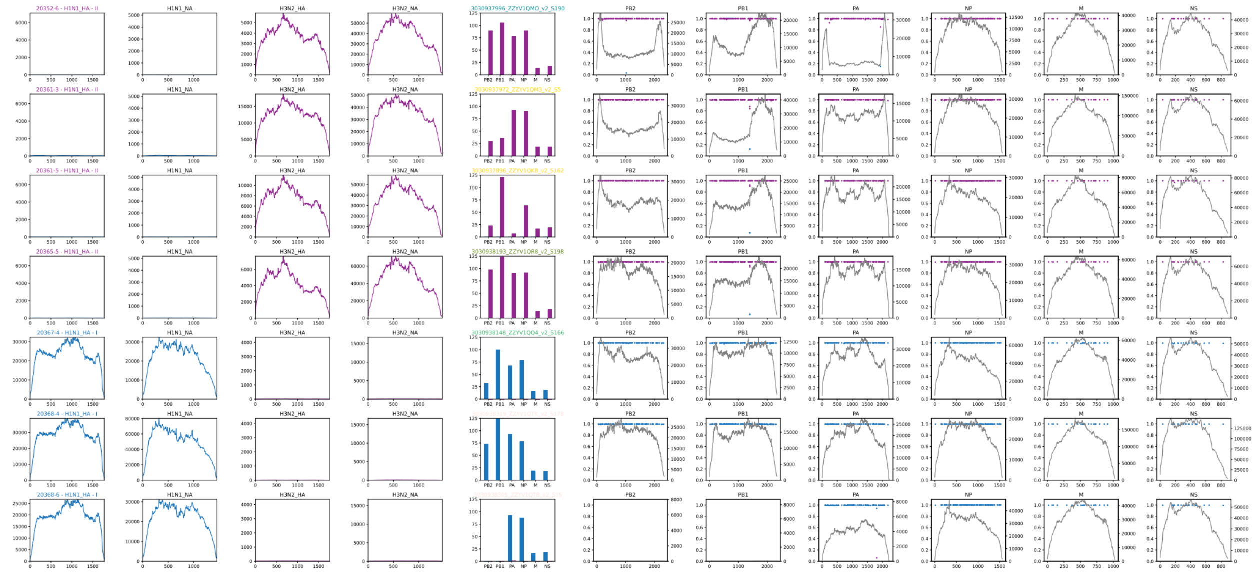

Figure S4. Coverage and the number of detected dSNPs for all samples, by segment. Each row is a separate sample. The pig ID, sample day, and sample classification are shown in the panel titles of the fifth column. The first four columns show mapping coverage against H1, N1, H3, and N2 references, respectively. The fifth column shows the number of dSNPs detected for each lineage in each of the six internal segments. The remaining six columns show coverage and dSNP frequency by location in the internal segments in the following order: PB2, PB1, PA, NP, M, NS. ***(Figure above is representative only, see attached PDF for full length figure).***
